# Song Perception by Professional Singers and Actors: An MEG Study

**DOI:** 10.1371/journal.pone.0147986

**Published:** 2016-02-10

**Authors:** Ken Rosslau, Sibylle C. Herholz, Arne Knief, Magdalene Ortmann, Dirk Deuster, Claus-Michael Schmidt, Antoinetteam Zehnhoff-Dinnesen, Christo Pantev, Christian Dobel

**Affiliations:** 1 Department of Phoniatrics and Pedaudiology, University Hospital Muenster, Muenster, Germany; 2 Institute for Biomagnetism and Biosignalanalysis, University of Muenster, Muenster, Germany; 3 German Center for Neurodegenerative Diseases (DZNE), Bonn, Germany; 4 Jean-Uhrmacher-Institute for Clinical ENT-Research, University Hospital Cologne, Cologne, Germany; 5 Department of Otorhinolaryngology, Friedrich-Schiller University Jena, Jena, Germany; UNLV, UNITED STATES

## Abstract

The cortical correlates of speech and music perception are essentially overlapping, and the specific effects of different types of training on these networks remain unknown. We compared two groups of vocally trained professionals for music and speech, singers and actors, using recited and sung rhyme sequences from German art songs with semantic and/ or prosodic/melodic violations (i.e. violations of pitch) of the last word, in order to measure the evoked activation in a magnetoencephalographic (MEG) experiment. MEG data confirmed the existence of intertwined networks for the sung and spoken modality in an early time window after word violation. In essence for this early response, higher activity was measured after melodic/prosodic than semantic violations in predominantly right temporal areas. For singers as well as for actors, modality-specific effects were evident in predominantly left-temporal lateralized activity after semantic expectancy violations in the spoken modality, and right-dominant temporal activity in response to melodic violations in the sung modality. As an indication of a special group-dependent audiation process, higher neuronal activity for singers appeared in a late time window in right temporal and left parietal areas, both after the recited and the sung sequences.

## Introduction

Recent research has increased our knowledge about the organization of neuronal networks for speech and music perception and suggests the presence of training-induced and interdependent modulation of musical and speech abilities [1]. This research is based on many studies on brain morphology, training effects and receptive/expressive functions of music and speech processing comparing instrumental musicians to novices [2–5]. In contrast, there is little knowledge about training or profession-specific cortical processing of speech and music as, for example, in professional voice experts such as actors in comparison to opera singers. Both of these groups have comparable levels of voice training and practice on stage, but with different emphasis on the specific type of voice training. Therefore, in order to investigate the training-related effects on brain function, it is much more informative to compare behavioral and neurophysiological data with respect to these fields of expertise, than to relate these groups to untrained novices. We regard singers and actors as a unique group of artists for comparative purposes, because both need to work on artistic expression through their voices as well as bodies and with high demands on self-perception. Furthermore, the comparison of these groups, using both a spoken and sung stimulus modality, addresses with very high specificity the questions of modality dependence, group dependence or any interaction in processing linguistic and musical content. Based on similar semantic and syntactic rule systems in language and in music, a complex and intertwined cerebral network for language and music processing is assumed [3,5–7]. Nevertheless, there is to date no study comparing two groups of experts who developed their expertise with very similar amounts and types of training with the same stimulus material, once in a sung and once in a recited modality.

### An intertwined network for processing music and language

Previous research transferred experimental approaches, which were established to investigate different levels of language processing, into the field of music processing. Most notable were designs employing semantic and syntactic expectancy violations. Semantic expectancy violations in language result in a N400 component generated mainly in the left superior temporal lobe, as evidenced by electro- and magnetoencephalographic (EEG and MEG) studies [8–14]. Syntactic violations in spoken sentences are reflected in an early negative electrophysiological component (ELAN; early left anterior negativity) and/or a late positive centro-parietal component (P600) over left anterior temporal and left inferior frontal regions [15]. Early left anterior magnetic fields after syntactical violation were also detected by MEG [16], while there is to date no clear correlate for the electrophysiological P600 component in MEG. Similarly to these findings, semantic and syntactic expectancy violations in musical material elicit negative electrophysiological components in right anterior frontal and superior temporal regions that are homologous to the above-mentioned left-lateralized speech-related correlates [17,18], i.e. the regions are highly similar, but with different hemispheric dominance. In a magnetoencephalographic study, high neuronal activity after musical syntactical violation was found in temporal regions on both hemispheres [19]. Typical “language” regions seemed to be less language-specific than previously thought [20]. Still, assuming a relative dominance of hemispheres in musical versus linguistic contexts, right temporal areas are reported to be mainly involved in processing and analyzing musical sequences [21–23]. In this vein, several studies investigated pitch violations in music and speech. An increment or decrement of fundamental frequency (final pitch) at the end of a spoken or sung line may represent a prosodic or melodic violation, respectively. Both can be interpreted as a violation of the syntactical rule system, and thus several studies focused on such prosodic/ melodic differentiations. They found evidence for positive centro-parietal and temporal components peaking between 300–600 ms after stimulus onset, as described for syntactical violations [24,25]. The amplitude of these components depended on the strength of violation (weak or strong) and on the degree of musical education of the participant [26,27].

In order to test whether simultaneously presented linguistic parameters (represented by semantic violations) and musical parameters (represented by melodic pitch violations at the end of a sung melody line) are processed dependently or independently, a medium is required that combines these two aspects. Comparing different musicians and laymen, Bonnel and coauthors [28] prepared excerpts from French operatic songs by manipulating the final word in such a way that it was either semantically congruous (S+) or incongruous (S-) and/ or by manipulating the final pitch of the melody line either in (P+) or out of key (P-). The simultaneous appearance of both an N400 and a P600 component in response to the combined prosodic and semantic violated condition (S-P-) suggested that semantic and syntactic aspects of language and music were processed by independent systems and did not compete for the same pool of mental resources in musicians and nonmusicians [28,29]. However, successive studies failed to find this division of labour, and rather presented evidence for more intertwined neuronal networks in bilateral middle and superior temporal gyri as well as inferior and middle frontal gyri, during combined musical and linguistic tasks [18,30,31]. Most of the above-mentioned studies compared the neurophysiological influence of linguistic content in divided sets of stimuli for language and music conditions, respectively. The advantage of using song lines performed by the human voice is that both linguistic and musical information are merged into one ecologically valid acoustic signal. The separation into a recited and a sung version allows a comparison of more linguistically-based and a more musically-based context with the same experimental material, which is a prerequisite for a study of highly professional artists. If there are interactions of semantic and syntactic processing in either a recited or sung modality, professional opera singers, as highly trained musical voice users, and professional actors as highly trained linguistic voice users, represent ideal subjects to search for neurophysiological correlates.

### Cognitive and neuronal characteristics for singers and actors

During singing, professional singers display increased activation of bilateral primary somatosensory cortex (where cortical representations of the larynx are situated), inferior parietal lobe and dorsolateral prefrontal cortex, and at a subcortical level, increased activation in the basal ganglia, thalamus and cerebellum compared to nonmusicians. This is is generally interpreted as evidence for training-induced cortical plasticity [32,33]. To the best of our knowledge, there is only one study investigating training-induced plastic effects as a result of acting training, identifying high activation during speech perception in bilateral premotor regions that are commonly activated by mouth movements [34].

Regarding a specialization of higher-order cognitive skills, several findings over the last few years point towards an enhanced quality of auditory imagery in musicians [35,36]. Musical imagery preserves many structural and temporal properties of auditory stimuli and can facilitate auditory discrimination by, for instance, the integration of semantically interpreted information and expectancies [37]. A special form of imagery, so called “audiation”, is described as an internal analog of aural music perception [38] and interpreted as a mental representation of music by internally “hearing” a music sequence that has just been auditorily or visually presented. It represents an integration of auditory, visual and/ or motor imagery in the brain and results in a cross-modal encoding of a unisensory input [39]. In line with such a description, audiation should be especially developed in musicians. However, the neural correlates of audiation haven’t been investigated so far.

### Aim and approach of the current study

The aim of our study was to investigate music and speech perception by voice experts, professional singers and actors, in order to disentangle the training-induced cortical networks for processing music and speech. To measure brain activity we used magnetoencephalography (MEG) due to its high sensitivity to time and its moderate to high accuracy in determining the underlying sources of brain activity [40]. This is the first study comparing these groups by using complex, but ecologically valid stimulus material in recited and sung modalities. Although all native-speaking participants are per definition linguistically highly educated in speaking their mother tongue, we consider it important to compare singers with actors in order to control for long-term voice training. This would be not the case in participants without such experience. To stimulate at a high artistic level, we used rhyme sequences of German art songs by Franz Schubert. Importantly, the lyrical basis for these songs is similar in structure to material that actors recite in a dramatic performance. One characteristic of art songs is a close integration of music and lyrics, typically without singing several notes in one syllable, a frequent feature in operatic arias. Since the songs are based on poetry, it is feasible to present the material both in a spoken and in a sung condition, thus comparing modality-specific processing of semantic and syntactic aspects.

Based on the nature of the semantic and melodic/ prosodic violations and because of using a sung and spoken modality, we expected increased activity upon violations in temporal areas in both hemispheres. Additionally, we predicted higher sensitivity for melodic/prosodic violations in singers and vice versa for semantic violations in actors. If singers indeed display more long lasting representations of auditory stimuli after their offset (i.e. what was above called audiation), we expect long-lasting activity in temporal regions, possibly with a right-hemispheric dominance, due to musical training.

## Material and Methods

### Participants

Fifteen professional singers (mean age = 29.2 years; 8 female) and 15 professional actors (mean age = 32.4 years; 9 female) took part in the experiment. The singers and actors had passed a university final qualifying examination after at least 4 years of training. At the time of the study, they currently practiced singing or acting on stage or in rehearsal for a minimum of 4 hours a day. The actors had not received any additional musical education besides compulsory music classes in high school and the singers had received articulation training for one year at the beginning of their university studies.

As an inclusion criterion all participants were familiar with the German art song cycles “Beautiful Miller Girl” and “Winter Journey” by the composer Franz Schubert, but had not practiced or performed them in auditions or on stage. All participants were right handed, free of neurological or psychiatric disorders, native speakers of German and had normal hearing thresholds as assessed by clinical audiometry. All gave written consent to participate in the study. The study protocol was approved by the local ethics committee of the Medical Faculty. The study was conducted according to the Declaration of Helsinki.

### Stimulus material

**Fig 1:**
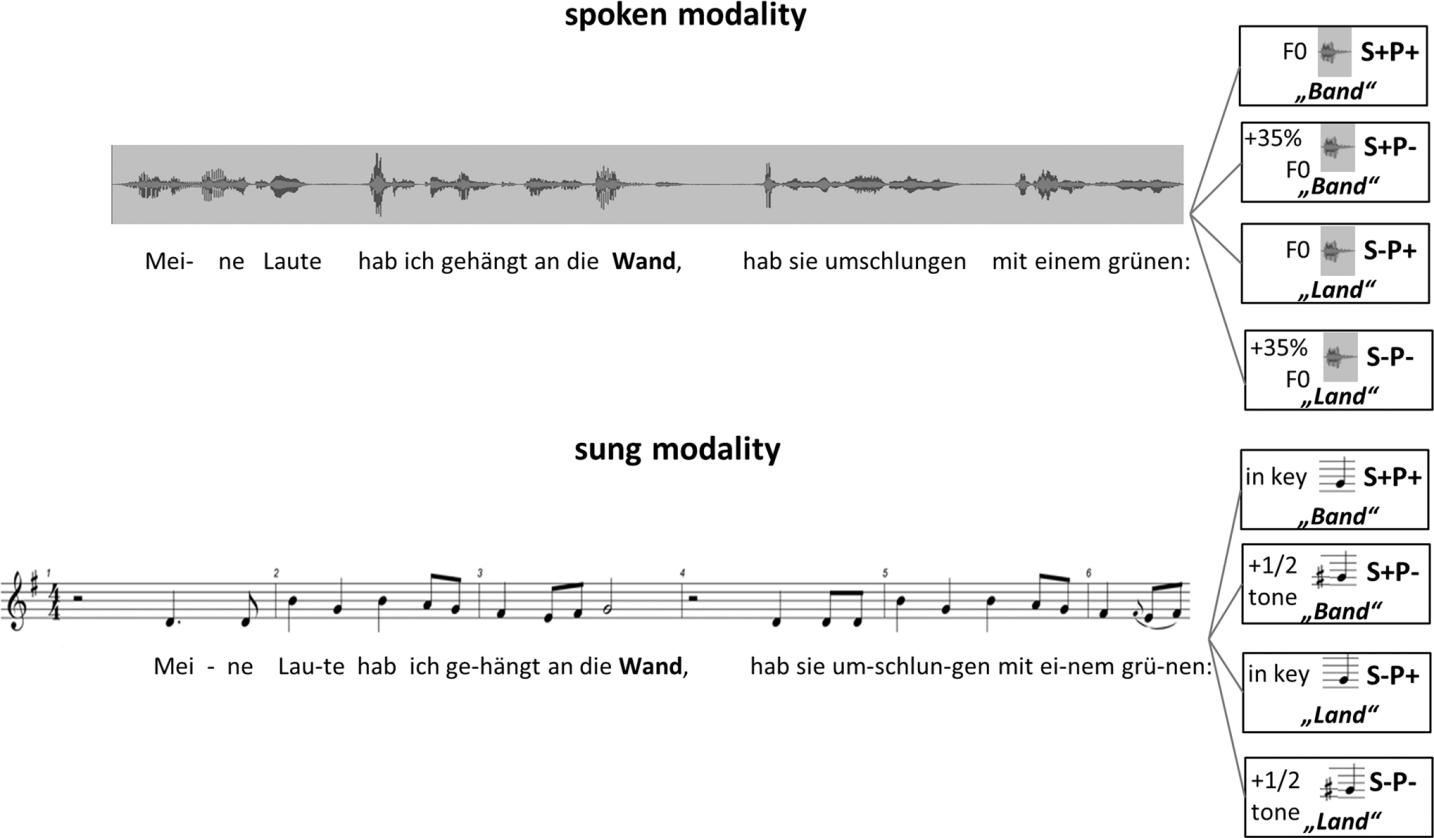
Example of a varied rhyme-couplet. Example of a varied rhyme-couplet from the song cycle “Beautiful Miller Girl” by Franz Schubert, poems by Wilhelm Müller: *Meine Laute hab ich gehängt an die* ***Wand***, *hab sie umschlungen mit einem grünen* ***Band*** (semantic correct) / ***Land*** *(incorrect)*, (english translation by Emily Ezust: My lute I´ve hung upon the wall, I´ve tied it there with a green **band/ land**). Semantic variation of the last word and/ or prosodic/ melodic variation of the final pitch resulted in 4 different conditions (S+: correct semantic sense, S-: incorrect semantic sense, P+: correct fundamental frequency/ final pitch, P-: incorrect fundamental frequency/ final pitch) for both spoken and sung modalities.

As indicated above, we used 30 short excerpts of songs from the romantic epoch (music by the German composer Franz Schubert, lyrics by Wilhelm Mueller) from the cycles “Beautiful Miller Girl” and “Winter Journey” for stimulation in the experiment [41,42]. The excerpts consisted of a rhyming couplet with a monosyllabic ending and the original melody line composed by Franz Schubert. For all excerpts, one version sung *a capella* (without accompaniment) and one recited, spoken version were recorded using a high-quality recording system and microphone (lingwaves software/ Wevosys 2010; microphone: 322 Datalogger, MK:216/ Voltcraft). For the recording, the same professionally educated singer sang and recited all excerpts. The duration of sung phrases ranged from 4.5 to 6.8 seconds (mean average 5.7 sec.), and the duration of recited phrases ranged from 4.2 to 5.4 seconds (mean average 4.8 sec.). In the same way, the mean length of the recited last words (452 ± 26 ms) differed from the mean of length of sung last words (710 ± 44 ms). For each modality (sung and spoken), the 30 excerpts were presented in four different conditions, resulting in 120 stimuli per modality. In the first condition, the original line was presented in the correct sung/ recited version (S+P+, for correct **s**emantic and **p**itch information). In the second condition, the pitch of the last word was decreased or increased in the sung modality by a semitone out of key in compliance with the original melodic contour (melodic violation), and in the spoken modality, by an increase of fundamental frequency of 35% (prosodic violation), which represents a violation of the expected decrease of prosody for a clause of statement (S+P-). A different relation between the deviation of a fundamental frequency in music and speech was first claimed by Besson et al. [27]. The authors described the deviation of 1/5 tone in music and 35% increase of the fundamental frequency (quart interval) in speech to be appropriate for a “weak” incongruity, because it is much harder to recognize such a difference in speech compared to the harmonic context of music. This is probably due to the strong harmonic rule system for melody compared to the only sensational rule system for speech prosody. After piloting our stimulus material with a group of healthy musical students, we decided an interval of ½ tone compared to 35% increase of fundamental frequency in speech to be more appropriate for our study. In the third condition, the original last word of the excerpt was exchanged by a semantically incongruent word (S-P+). These semantically incongruent monosyllabic words fulfilled the original rhyme scheme. In the fourth condition, we presented a double incongruency at the end of the excerpt with an incorrect pitch ending (syntactic/ prosodic violation) and a semantically incongruent last word (S-P-). All pitch manipulations in the sung and spoken modality were performed on the original, digitally stored sound files using the software PRAAT (Version 5.3.34) to ensure the correct pitch violation (Fig 1).

### Procedure

Subjects were comfortably seated in a magnetically shielded room and their head position was stabilized in the MEG scanner using soft pads. All stimuli were presented binaurally 60 dB above the individual hearing threshold of each ear, which was determined at the beginning of the experiment with an accuracy of at least 5 dB by reduction of the sound of one stimulus sentence to the minimal individual sound level both for the sung and spoken modality. Instructions, visual prompts and feedback were presented via back-projection on a screen in front of the subject that was adjusted in height to be comfortably visible for the subject.

Subjects worked through the experimental instructions and eight practice trials at their own pace. Stimuli used for the practice trials were not used again in the subsequent experiment. The 240 stimuli were presented in 4 experimental runs per 60 stimuli using the software Presentation (Neurobehavioral Systems Inc., Albany, CA, USA). Within each run, stimuli were presented in a pseudo-randomized order. Occurrence of the different versions of each excerpt were distributed equally across four runs, with the constraint that two versions of the same excerpt did not occur subsequently and not more than 3 stimuli from the same condition were played consecutively.

After the presentation of each stimulus, subjects had to judge the accuracy of the semantic congruence of the last word and the accuracy of the pitch of the last word, both for sung and for spoken stimuli. Subjects responded by means of successive button presses and were visually prompted to give their responses, with the prompt for the first judgment appearing 1500 ms after stimulus offset. They were instructed to respond within 2000 ms. The next prompt or trial was presented automatically after the subject’s response or after a time lapse of 5 seconds. The order of the prompts (semantic and pitch judgments) and the assignment of buttons to responses (correct and incorrect) were balanced across participants and remained the same for each subject throughout the experiment. Each run took around 15 minutes and the entire measurement process including instructions, practice trials and pauses between runs, lasted about 90 minutes.

After the measurements, subjects took part in a semi-structured interview to summarize how attention-demanding they had found the tasks to be. For evaluation purposes, the answers were classified into three different categories from “low”, “moderate” and “high” level of attention.

### MEG recordings and data analysis

MEG signals were recorded continuously, using a whole-head device with 275 first-order axial SQUID gradiometers (Omega 275, CTF, VSM MedTech, Coquitlam, Canada), filtered online (150 Hz low-pass for aliasing, 50 Hz notch for European power grid) and sampled at 600 Hz. The continuous data were then band-pass filtered offline in a 0.1–48 Hz range, using a zero-phase second-order Butterworth filter. The triggers for data analysis were set at the beginning of the last word for each stimulus. For each trial, epochs ranging from 200 ms before acoustic trigger at the word onset to 2000 ms after onset were extracted from the continuous data. Artifact rejection and pre-processing, with baseline correction of the first 100 ms and rejection of sensor activity higher than 3000 fT, was performed with EMEGS 2.3 [43] running under MATLAB 7 SP3 (The MathWorks, Natick, MA, USA). Epochs for each condition were averaged. Individual averages were standardized on the mean MEG sensor configuration across all participants and runs, and thus corrected for differing head positions of the participants within the MEG scanner. The amplitude and distribution of event-related magnetic fields depended on the individual head position within the sensor coordinate system, as well as individual head geometry, especially head size. An estimation of the underlying neuronal generators, such as the L2- Minimum-Norm Estimate, (L2-MNE; [40] however, is independent of such individual factors and enables statistical tests across participant groups and conditions. The L2-MNE served as an inverse-distributed source modeling method for examining the cortical generator of the magnetic field activity without a priori assumptions about the location and/or number of current sources. The present analyses were based on an isotropic spherical head model with 197 dipolar sources evenly distributed on an inner spherical shell. The sphere position and radius were estimated in order to optimally fit the digitized head shape of each participant. Across all participants and conditions, a Tikhonov regularization parameter of k = 0.2 was applied.

Dipole strength at a given dipole site was obtained as the square root of the sum of squared L2 values for each of the two tangential orientations, for each time point for each data set. The L2-MNE amplitudes were analyzed with a point-wise repeated measures ANOVA with the within-subject factor CONDITION and the between-subject factor GROUP separately for the spoken and sung modalities. To avoid false positives, a significance criterion of p<0.01 was used, and significant effects were considered only when they were observed for at least 10 consecutive sampling points (e.g. around 15 ms) and at least 10 neighboring dipoles. The statistical parametric F values were mapped on a standard cortical surface in time slots of 50 ms in order to display the origin of effects in more detail. Such foci of high activity were further analyzed by averaging the mean activity as different clusters of both hemispheres. This type of analysis for multichannel recordings (EEG and MEG) has become an established procedure for sensor and source space (recent studies include [12,44].

For a comparison of local clusters with high activity, the relevant time windows for defining clusters were based on significant activation differences of the point-wise ANOVA. In line with the literature, we detected clusters of activity in an interval between 200 and 500 ms after onset of the last word in both temporal lobes, and we described foci of activity in this time window as “early” components. Because we also detected activation peaks in a second interval between 600 and 1700 ms with a significant dependence on the between-factor GROUP, we described these activations as “late” components. Even though we defined the clusters in a data-driven manner, the dipoles in these clusters overlapped substantially comparing the hemispheres separately for each modality (spoken modality: left hemisphere 22 dipoles and right hemisphere 22 dipoles, with 18 corresponding dipoles; sung modality: left hemisphere 16 dipoles and right hemisphere 19 dipoles, with 11 corresponding dipoles). For “early” cluster comparison, we calculated a repeated measures ANOVA including within-subject factors SEMANTIC VIOLATION, MELODIC/ PROSODIC VIOLATION, HEMISPHERE and between-subjects factor GROUP. Because no corresponding dipoles were found in the “late” clusters, we calculated separately for each hemisphere a repeated measures ANOVA, including within-subject factors SEMANTIC VIOLATION, MELODIC/ PROSODIC VIOLATION and between-subject factor GROUP.

All analyses were conducted separately for the sung and spoken modality to minimize bias caused by differences in length of the last word for the sung and spoken versions and the different time window of resulting magnetic fields. Pairwise post-hoc comparisons between significant and relevant condition pairs were computed and thresholded by Bonferroni correction.

### Analysis of behavioral data

In order to evaluate the behavioural data, we computed the mean values of hits and correct rejections concerning melodic/ prosodic correctness of the last word as the accuracy of pitches and concerning the semantic congruence of the last word, as the accuracy of words, in order to obtain more detailed information of the kind of mistakes associated with the different conditions. In the same way as for the MEG data, performance scores were analyzed using a repeated measures ANOVA with the factors CONDITION and GROUP separately for the sung and spoken modalities and additionally, as mentioned before, each for accuracy of pitches and accuracy of words. The ANOVA results are reported when significant at p < = 0.05. All p values for results were adjusted, when necessary, with the Greenhouse-Geisser epsilon correction for nonsphericity. Pairwise post-hoc comparisons between significant and relevant condition pairs were computed and thresholded as before by Bonferroni correction.

## Results

### Behavioural data

#### Spoken modality

**Fig 2:**
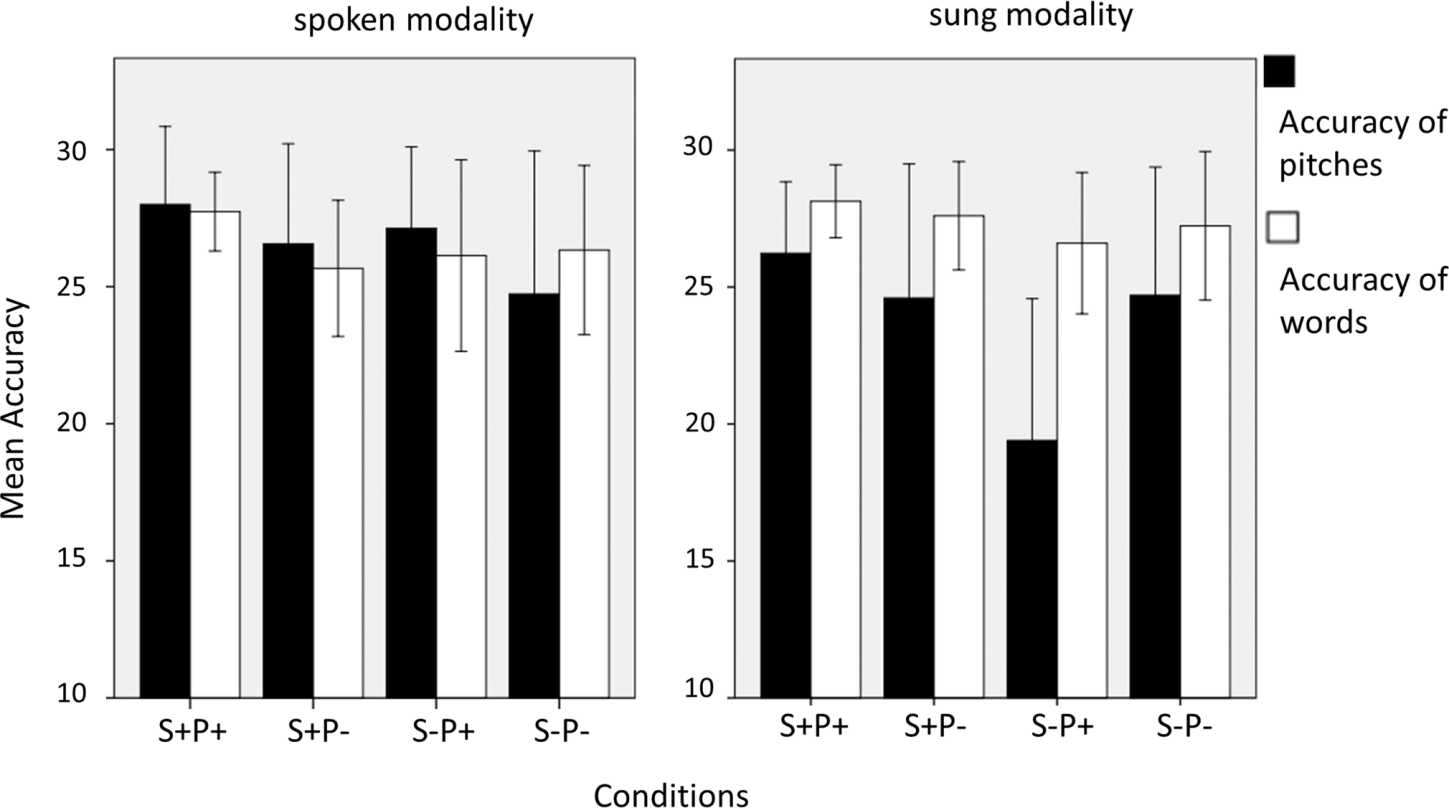
Mean values of accuracy of pitches and accuracy of words. Mean values of accuracy of pitches and accuracy of words (max. 30) for all conditions (S+: correct semantic sense, S-: incorrect semantic sense, P+: correct fundamental frequency/ final pitch, P-: incorrect fundamental frequency/ final pitch) for both spoken and sung modalities. Error bars indicate one standard deviation.

The results concerning the accuracy of the ending pitch of the line (melodic/ prosodic violation) revealed a significant main effect of CONDITION (F_(1.75, 49.11)_ = 6.33, p = .005), and no main effect of and interaction with GROUP. Comparing the significant mean values for post-hoc analysis (Fig 2) showed that the performance was lowest for the condition with double incongruency S-P- (24.7 ± 5.2 correct responses). This condition differed from all other three (S+P-: 26.5 ± 3.6 c.r., S-P+: 27.1 ± 2.9 c.r. and S+P+: 28.0 ± 2.8 c.r.; post-hocs: S+P+ vs. S-P-, p = .003; S+P- vs. S-P-, p = .011; S-P+ vs. S-P-, p = .020), but they were not significantly different from each other.

Identifying the semantic accuracy of the last words (accuracy of words) in the spoken modality yielded a significant main effect CONDITION (F_(1.69, 47.36)_ = 4.24, p = .026), and again, no main effect of and interaction with GROUP. Comparing the significant mean values for post-hoc analysis showed that the performance was nearly the same for all conditions that contained an expectancy violation (S+P-: 25.6 ± 2.4 c.r., S-P+: 26.1 ± 3.4 c.r., S-P-: 26.3 ± 3.0 c.r.), but they all yielded lower accuracy compared to the correct recited line (S+P+: 27.7 ± 1.4 c.r.; post-hocs: S+P+ vs. S+P-, p < .001; S+P+ vs. S-P+, p = .024; S+P+ vs. S-P-, p = .029).

#### Sung modality

**Fig 3:**
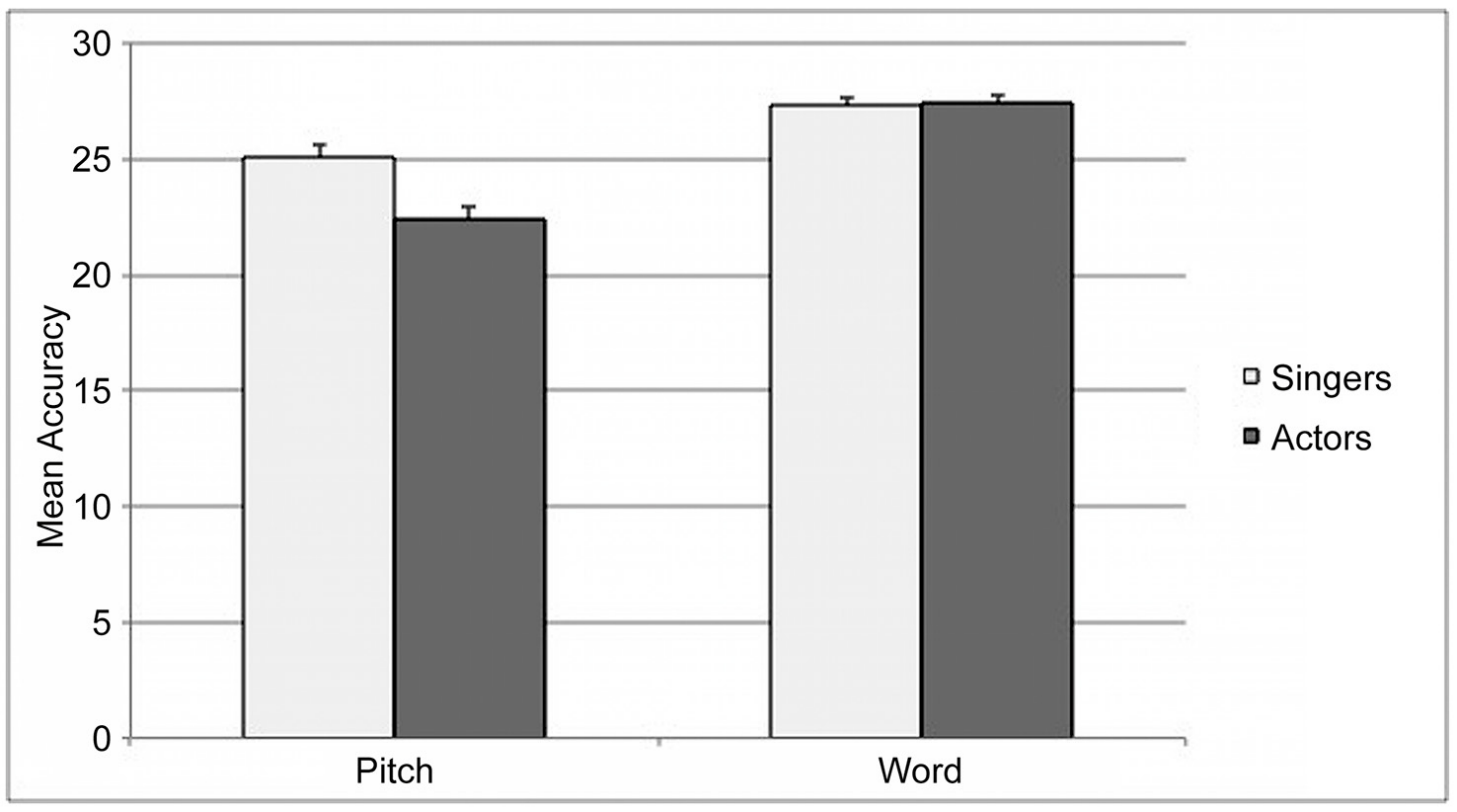
Mean values of accuracy of pitches and words in the sung modality. Mean values of accuracy of pitches and accuracy of words (max. 30) in the sung modality in comparison of singers and actors. Error bars indicate the standard error.

In the sung modality, the singers reached a significantly higher accuracy regarding the judgment of pitches (25.0 ± 1.3 c.r) compared to actors (22.4 ± 1.6 c.r.; main effect GROUP: F_(1, 28)_ = 11.05, p = .002) without an interaction with CONDITION (Fig 3). Also, we found a significant main effect of CONDITION (F_(1.57, 44.14)_ = 14.33, p < .001). The post-hoc analysis (Fig 2) revealed a significant difference in recognizing the correct pitch combined with a semantic violation (S-P+: 19.4 ± 5.1 c.r.) compared to the other conditions (S+P-: 24.6 ± 4.8 c.r., S-P-: 24.7 ± 4.6 c.r., S+P+: 26.2 ± 2.6 c.r.; all post-hoc comparisons with S-P+ ≤ .001).

The accuracy of semantic sense of the last word in the sung modality again showed a significant main effect CONDITION (F_(1.96, 54.88)_ = 3.19; p = .048) with no interaction with or main effect of GROUP. The post-hoc analysis of the main effect CONDITION (Fig 2) confirmed the S-P+ condition as most difficult to recognize in the sung modality (26.6 ± 2.5 c.r.), differing significantly from the correct line S+P+ (28.1 ± 1.3 c.r.; S-P+ vs. S+P+, p < .007) and differing in terms of non-significant trends and from S-P- (27.3 ± 2.7 c.r.) and from S+P- (27.6 ± 1.9 c.r.; S-P+ vs. S+P-, p = .051; S-P+ vs. S-P-, p = .054).

### Group differences with regard to attention required and fatigue

We observed differences between singers and actors at the level of attention reported necessary to perform successfully. Summarized in semi-structured interviews after the measurements, subjects were categorized according to three different levels of effort. In the group of actors, nine subjects reported the need to pay substantial attention during the experiment and felt exhausted at the end. Four subjects described the required attention as “moderate” and two as “low”. In contrast, ten singers reported a kind of “easy-flow” and a “inner rehearsal in the mind” described also in terms of internal repetition of the spoken and sung lines with focus on melodies but also on texts and no need for additional attention. These ten singers reported low attention demands, while four other singers reported a moderate level and one a high level.

### Magnetoencephalographic data

#### Global power

**Fig 4:**
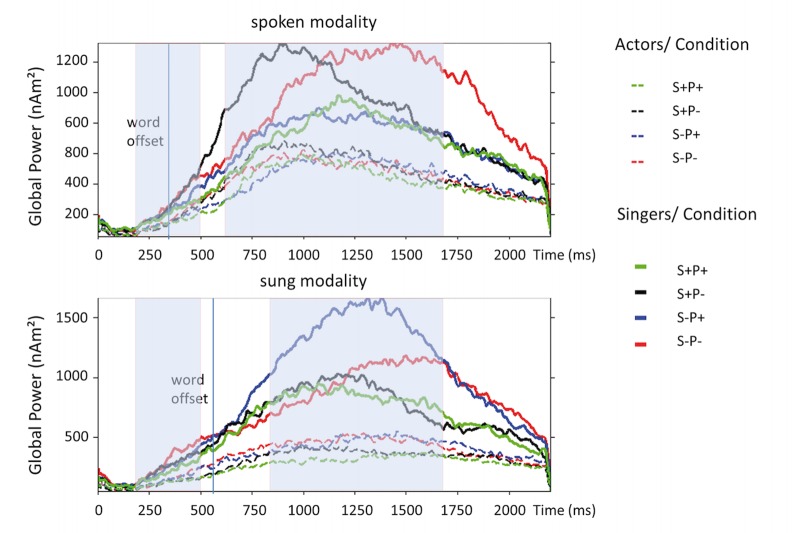
Global power of minimum norm estimates of all dipoles. Global power of minimum norm estimates of all dipoles, separated for the different conditions, for singers and actors, and for both spoken and sung modalities. Blue shadowed are the time windows of the analysis of the early and late activity.

Inspection of the Global Power of L2-MNE solutions (Fig 4) demonstrated a long interval of high activation, starting around 400 ms after last word onset/ peaking around 1000 ms for spoken stimuli and starting around 600 ms/ peaking around 1250 ms for sung stimuli, with much higher cortical activity for singers than for actors.

The results of the point-wise repeated measures ANOVA revealed two time windows for both modalities, in which the experimental manipulations resulted in different brain responses. Anticipating briefly the statistical results below, during the early time window (200–500 ms), brain responses differed in predicted ways between the violations, but independent of group. In the late time window (600–1700 ms), no difference between conditions was found, but singers displayed a unexpected long-lasting and substantial level of activity. Because the topography of evoked activity was rather stable and varied only minimally within these time windows, we will present only averaged responses for these intervals.

### Statistical analysis of the early activity

**Fig 5:**
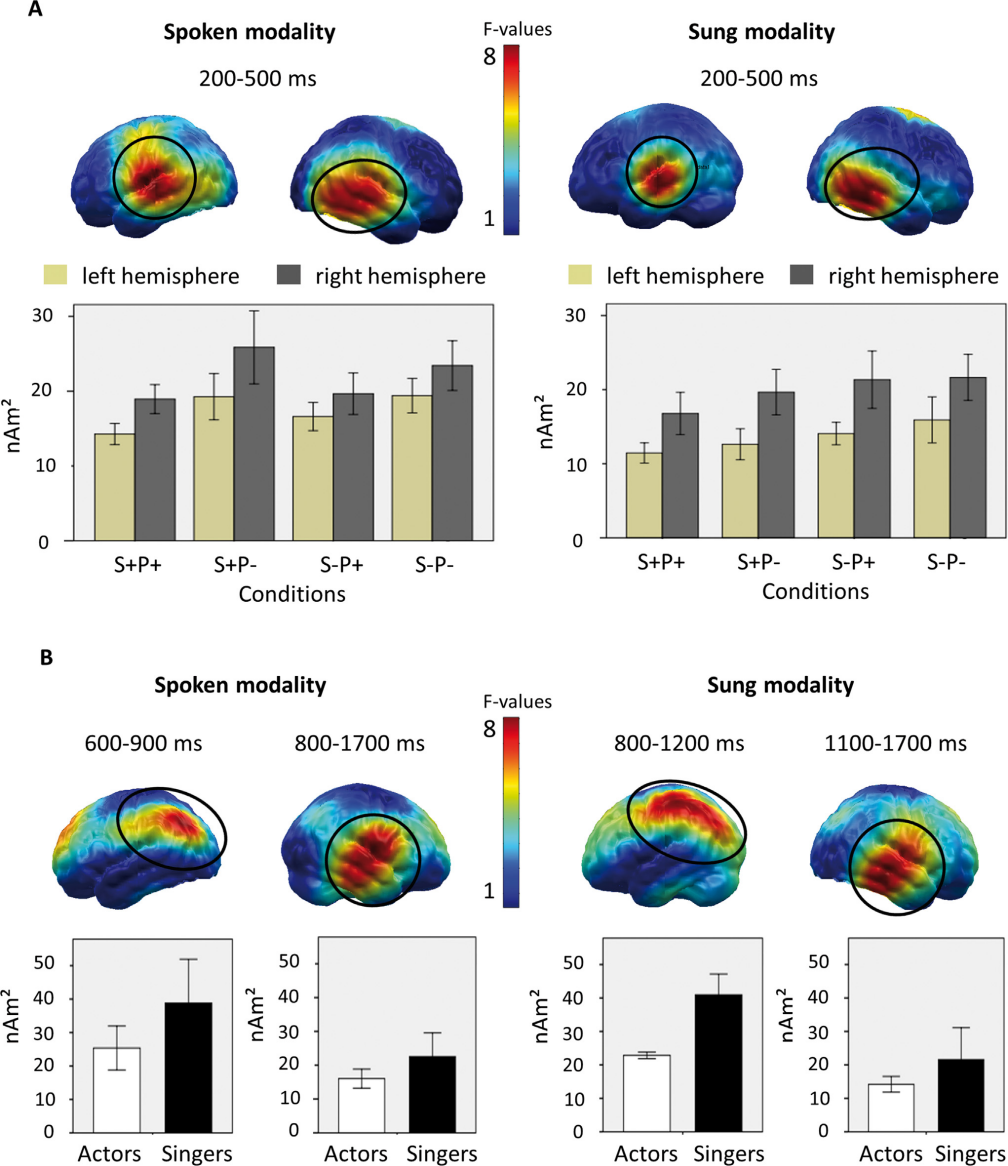
Maps and mean neural activity for respective clusters of dipoles. A. Top: Mapping of the F-values for the interaction CONDITION and HEMISPHERE on a cortical surface for average time intervals, for both spoken and sung modalities. Bottom: Mean neural activity for the respective clusters of dipoles for all conditions and for both spoken and sung modalities. Error bars denote one standard deviation. B. Top: Mapping of the F-values for the main effect group on a cortical surface for average time intervals, for both spoken and sung modalities. Bottom: Mean neural activity for the respective clusters of dipoles for both groups and for both spoken and sung modalities. Error bars denote one standard deviation.

The pointwise repeated measures ANOVA revealed that the factors SEMANTIC VIOLATION and PROSODIC (spoken modality)/ MELODIC (sung modality) VIOLATION differed significant in the left and right temporal regions, averaged for the time interval of 200–500 ms (Fig 5). Because of corresponding dipole groups in the temporal areas of both hemispheres, we included the factor HEMISPHERE in the analysis of early activity. For both modalities, no significant statistical effects were detectable for the between-subject factor GROUP or for any interaction with GROUP according to the thresholds described in the methods section.

#### Spoken modality

Repeated measures ANOVAs for both temporal clusters (left temporal: 200-500ms, 22 dipoles; right temporal: 200–500 ms, 22 dipoles; with 18 corresponding dipoles) revealed significant main effects of the factor HEMISPHERE (F_(1,28)_ = 14.73, p = .001), representing higher activation on the right hemisphere (21.97 ± 5.45 nAm^2^) than on the left hemisphere (17.39 ± 4.97 nAm^2^). Furthermore, there was a main effect of the mean activity of PROSODIC VIOLATION (F_(1,28)_ = 32.97, p < .001) comparing prosodic violations (S+P- and S-P-: 21.99 ± 6.44 nAm^2^) to conditions without violation (S+P+ and S-P+: 17.38 ± 5.77 nAm^2^), but no effect of SEMANTIC VIOLATION. The only significant interaction SEMANTIC VIOLATION X HEMISPHERE (F_(1,28)_ = 65.76, p = .007) reflected higher activity for conditions with semantic violations (mean of: S-P+ and S-P-: 18.01 ± 5.46 nAm^2^), compared to conditions without semantic violations (mean of: S+P+ and S+P-: 16.77 ± 5.48 nAm^2^) on the left hemisphere (t_(29)_ = 3.35, p = .002) and the opposite pattern (mean of: S-P+ and S-P-: 21.54 ± 7.93 nAm^2^; mean of: S+P+ and S+P-: 25.86 ± 13.08 nAm^2^) on the right hemisphere (t_(29)_ = -3.35, p = .003).

Thus, for the spoken modality, there was generally higher activity on the right hemisphere. Both hemispheres were equally involved in the processing of prosodic violations, while there was a hemispheric specialization for processing semantic violations. For the latter, the left hemisphere displayed stronger activity as a response to violated sentences than correct sentences, i.e. a typical N400 effect.

#### Sung modality

Repeated measures ANOVAs for both temporal Clusters (left temporal: 200-500ms, 16 dipoles; right temporal: 200–500 ms, 19 dipoles; both hemispheres: 11 corresponding dipoles) revealed significant main effects of the factor HEMISPHERE (F_(1,28)_ = 34.23, p < .001) representing higher activation on the right hemisphere (19.86 ± 4.43 nAm^2^) than on the left hemisphere (13.52 ± 3.77 nAm^2^). Moreover, a significantly higher level of activity was detected for expectancy-violated conditions than those without MELODIC VIOLATION (F_(1,28)_ = 10.17, p = .004; S+P- and S-P-: 17.46 ± 3.24 nAm^2^ vs. S+P+ and S-P+: 15.91 ± 4.13 nAm^2^) and SEMANTIC VIOLATION (F_(1,28)_ = 29.17, p < .001; S-P+ and S-P-: 18.25 ± 3.42 nAm^2^ vs. S+P+ and S+P-: 15.13 ± 2.81 nAm^2^). These main effects were modulated by the significant interaction SEMANTIC VIOLATION X MELODIC VIOLATION X HEMISPHERE (F_(1,28)_ = 39.44, p = .014), which themselves resulted from a significant interaction of SEMANTIC VIOLATION X MEDOLDIC VIOLATION only on the right hemisphere (F_(1,29)_ = 49.71, p = .010), not present on the left hemisphere (F_(1,29)_ = 1.29; n.s.). Post-hoc analysis for this interaction revealed a similar level of activity for the conditions with a semantic violation (S-P+: 21.34 ± 3.19 nAm^2^, S-P-: 21.65 ± 4.32 nAm^2^), while there significant difference emerged in comparing the original line (S+P+: 16.78 ± 2.07 nAm^2^) to the condition that only contained a melodic violation (S+P-: 19.66 ± 3.11 nAm^2^; t_(29)_ = -3.98, p < .001).

Thus, for the sung modality, there was generally more activity on the right hemisphere. Both hemispheres responded to semantic and melodic violations, while there was an additional interaction of melodic and semantic violations only in the right hemisphere. Here, stronger activity was only found in response to a melodic violation, if there was no semantic violation.

### Statistical analysis of the late activity

The pointwise repeated measures ANOVA revealed, for both modalities, a significant main effect of the between-subject factor GROUP, representing higher neuronal activity for the singers than the actors localized in right temporal and left parietal regions in a late time window (Fig 5). There was neither a significant effect for the factors SEMANTIC VIOLATION and PROSODIC/ MELODIC VIOLATION, nor for any interaction with GROUP, according to the thresholds mentioned in the above methods section. The absence of corresponding dipole groups on the homologous hemisphere did not warrant the additional factor HEMISPHERE in the analyses.

#### Spoken modality

Repeated measures ANOVAs on time-averaged activity levels in the left parietal cluster (600-900ms, 10 dipoles) revealed a significant GROUP effect (F_(1,28)_ = 4.68, p = .039), with higher activity for the singers (41.11 ± 13.2 nAm^2^) than the actors (29.64 ± 5.82 nAm^2^), independent of the stimulus condition. Additionally, in the right temporal cluster (800-1700ms, 19 dipoles), singers displayed significantly higher activity (22.56 ± 5.21 nAm^2^) than actors (16.04 ± 2.34 nAm^2^; F_(1,28)_ = 11.20, p = .002).

#### Sung modality

Repeated measures ANOVAs on time-averaged activity levels revealed a similar pattern of results to the spoken modality. In the left parietal cluster (800-1200ms, 21 dipoles), we found a significant GROUP effect (F_(1,28)_ = 8.27, p = .008) with higher activity for the singers (40.94 ± 4.42 nAm^2^) than the actors (22.87 ± 0.91 nAm^2^). Additionally, for the right temporal cluster (1100-1700ms, 18 dipoles), we found significantly higher activity for the singers (21.58 ± 8.41 nAm^2^) compared to the actors (14.21 ± 2.14 nAm^2^; F_(1,28)_ = 8.44, p = .007).

## Discussion

The aim of this study was to compare linguistic and musical processing in two groups of highly trained voice users, i.e. professional singers and actors. We employed rhyme sequences from German art songs, and presented analogous semantic and/ or melodic/prosodic violations in sung and recited versions of the material. MEG measurements were implemented to identify functional brain activity with regard to the type of expertise. Behavioral data revealed greater accuracy of pitch detection in the sung modality for singers than for actors, while there were no detectable group-specific advantages for actors, neither for the sung nor for the recited material. Although previous studies referred to dependence in the neuronal processing of linguistic and musical dimensions, both in a spoken and sung modality [5,30,45], this is the first study presenting combinations of semantic and melodic/ prosodic expectancy violations for speaking and singing in a complex, but ecologically valid context. Confirming an intertwined neuronal network for music and speech, MEG data analysis disclosed condition- and modality-specific differences of “early” temporal activity (200–500 ms) on both hemispheres in homologous clusters, independent of the kind of expertise. Significant group differences appeared as “late” neuronal activity (600–1700 ms) for both stimulus modalities in right temporal and left parietal areas. We will discuss the results of the behavioural data and the two time windows in turn.

### Behavioural data

In the behavioral data, we did not find an effect of modality-specific expertise, apart from for higher accuracy for pitch discrimination in singers for the sung modality, which can be explained with a higher sensation for musical patterns in their familiarized domain. Interestingly, while performance in terms of word accuracy in the sung and spoken modality was nearly at the same level for the different conditions, both groups performed lower for the discrimination of correct pitches in the case of a semantic violation (S-P+) in the sung modality. This might result from the need for high attention to recognize the semantic sense of the words, at the expense of the discriminatory power of pitches. Due to the different pronunciation of speech in singing compared to speaking, vowels gain stronger emphasis than consonants. This might have created particularly challenging conditions for the discrimination of closely related phonemes, such as in the rhyme words that were used to replace the original words.

### Early neuronal activity related to linguistic and musical context

Summarizing the similarities of the early effects for the sung and spoken modalities, high neuronal activity was measured especially after melodic/prosodic violations in predominantly right temporal areas. Consequently, it seems that neuronal networks involved in processing both modalities exhibited higher neuronal activity for the expectancy violation of the final pitch deviation of the lines compared to semantic violations. Therefore, in the present design, the rule system for syntax—melodic/ prosodic aspect—represents a global characteristic for both sung and recited phrases and indicates a global syntactical system represented bilaterally with dominance of the right hemisphere if factors involfing pitch (melody, prosody) are violated (see e.g. [46]). In line with our findings, previous studies investigating linguistic aspects of speech revealed a dominance of left temporal areas [13,15,16], especially if linguistic stimuli were presented in a complex syntactical structure. In comparison, a dominance of right temporal areas was found after violation of a musical order system such as a chord sequence or a melody line [17,19,23,47]. This was the case if linguistic and musical tasks were performed by human voice, mixed-animal or vocally similar sounds demanding high attention for frequency analysis [48,49].

The above-mentioned similarities during the processing of recited and sung phrases are contrasted by different interactions of effects for the sung and spoken modalities, presenting a more complex dependence of information processing for semantic and prosodic/ melodic content on both hemispheres. While there was a predominantly left-temporal lateralized activity after semantic expectancy violations for the recited sequences, which is in line with findings for the classical N400 effect [50,51], this was opposed by right-dominant temporal activity in response to melodic violations, but semantic correctness, in the sung version. The combination of the semantic content with the musical syntactical form of the melody line seems to be strongly connected in the modality of singing and represents intertwined networks reacting to different degrees after expectancy violation. These findings confirm recent research revealing a more bilateral temporal network system dependent on modality-specific aspects for sung and spoken units [5,30,45]. But compared to these findings, the present study combined syntactical and semantic violations in a complete rhyme sequence both in a spoken and a sung modality. To the best of our knowledge, the only design using also original excerpts to compare different musicians and laymen (i.e. different romantic opera composers), but only presented in a sung modality, was presented by Besson, Schon and Bonnel [28,29]. In their study, the simultaneous violation of the semantic and the syntactic sense at the end of a sung line resulted in a N400 and a P600 component, suggesting that semantic and syntactic aspects of language and music were processed by independent systems, which was, however, not confirmed in subsequent investigations. In contrast, vocally generated stimuli which create simultaneously high demands on linguistic and musical aspects seem to involve middle and superior temporal areas, acting as an intertwined network. This network is adapted in a modality dependent way to different conditions [5,52–54].

### Group differences in late neuronal activity

During the analysis, we detected differences in brain activation in an unexpected late and long-lasting time window (up to 1700 ms after stimulus onset), with higher activation for singers than actors. The activity was localized in right temporal areas, similar to the early activation clusters generated by the semantic and syntactic incongruencies, as well as in parietal areas on the left hemisphere, which are known to be involved in higher-order music cognition [36].

The specific role of temporal areas on both hemispheres concerning speech and music processing was discussed before in the interpretation of “early” activation clusters. We interpret the renewed appearance of neuronal activity in the right temporal area as a special form of working memory function. In contrast to actors, in the semi-structured interviews, singers reported repeating a heard sequence in their minds (inner rehearsal). Thus, the late right temporal activity might stem from cognitive processes representing an illusionary perception of previously perceived auditory stimuli. Studies in the field of music psychology described findings of a mental representation of music in musicians, by internally hearing sounds after the onset of the physical stimulus [37,39], without any neurophysiological evidence of this phenomenon being reported so far. Right temporal lobe activity, as a correlate of a very vivid mental representation of music, is also supported by a recent fMRI study of musical imagery of familiar tunes. This study reported a relationship of activity in right secondary auditory areas and the subjective vividness of mental imagery [55]. Because we found the higher activity in musicians for both the sung and the spoken modality, we assume that this is the result of a transfer effect after extensive training. There is some evidence for the existence of two separate working memory functions in musicians for music (nonspeech) and speech material: a phonological loop and a tonal loop. Thus, training effects of the tonal loop seem to carry over to the phonological loop, involving highly similar neural correlates [56].

The functional role of the inferior parietal cortex involves auditory-verbal working memory and short-term memory for musical pitch, especially on the right hemisphere of musically trained subjects [57]]. More generally, the inferior parietal cortex has been associated with the integration of sensory and motor signals for the somatosensory guidance of movements [58]. Previous neuroimaging studies have documented its response to both speech and music perception [49]. A model of speech motor control [59] posits a role of the parietal cortex (PC) in a feed forward control mechanism of articulatory motor commands. In this model, the PC acts as a control system for somatosensory feedback from the vocal tract by comparing the actual kinaesthetic feedback with the expectation of the pronounced sound. Accordingly, we assume that the increment in PC activity could reflect enhanced processing of a mismatch between intention, action and consequences and thus allows for more rapid sensorimotor adaptions/ corrections in singers than actors. Another previous study presented evidence that increased activity of receptive systems subserve the precise transformation of highly automatic speech motor sequences into appropriately adjusted motor patterns for singing [32]. Kleber and coauthors demonstrated that imagined and overt singing involves partly different brain systems in singers, with imagined singing activating a large frontal and parietal network, indicating increased involvement of higher-order cognitive processes during mental imagery [60]. These topical findings during receptive and expressive functions supplement our findings and suggest an important role of the parietal cortex for music processing in singers. Additionally, it has been shown that complex mental transformations of musical material, like the mental reversal of imagined melodies, is related to activity in the posterior parietal cortex [36]. Performing music in the mind is a technique used by professional musicians to rehearse various aspects of a musical piece, for example to mentally revise difficult parts of a previously executed musical passage. As such our unexpected findings integrate well into the existing literature, but it is yet an open question whether the phenomenon of audition and its neurophysiological counterpart comes into existence through training or whether this is prerequisite to become a professional musician.

## Conclusions

In conclusion, our results of early and late neuronal activation are in line with studies emphasizing a bilateral neuronal network during linguistic and musical auditory processing, which can be tuned according to the level of mental demand [5,11,52,54,61]. Regarding the effect of experience, we did not find any early differences of neuronal activities evoked by a semantic and/or melodic/ prosodic violation. In contrast, rather late and long lasting time windows were characterized by strong activity in left parietal and right temporal areas. We propose that these are related to stronger mental imagery and higher-order music cognition in singers. This might constitute the effect of musical training or a prerequisite.
