# Correction: Song Perception by Professional Singers and Actors: An MEG Study

**DOI:** 10.1371/journal.pone.0154549

**Published:** 2016-04-25

**Authors:** The PLOS ONE Staff

The seventh author’s name is spelled incorrectly. The correct name is: Antoinette am Zehnhoff-Dinnesen. The publisher apologizes for the error.

## References

[1] Rosslau K, Herholz SC, Knief A, Ortmann M, Deuster D, Schmidt C-M, et al (2016) Song Perception by Professional Singers and Actors: An MEG Study. PLoS ONE 11(2): e0147986 doi: 10.1371/journal.pone.0147986 26863437 10.1371/journal.pone.0147986 PMC4749173

